# Closed-Loop and Robust Control of Quantum Systems

**DOI:** 10.1155/2013/869285

**Published:** 2013-08-07

**Authors:** Chunlin Chen, Lin-Cheng Wang, Yuanlong Wang

**Affiliations:** ^1^Department of Control and System Engineering, Nanjing University, Nanjing 210093, China; ^2^School of Physics and Optoelectronic Technology, Dalian University of Technology, Dalian 116024, China; ^3^Institute of Cyber-Systems and Control, State Key Laboratory of Industrial Control Technology, Zhejiang University, Hangzhou 310027, China

## Abstract

For most practical quantum control systems, it is important and difficult to attain robustness and reliability due to unavoidable uncertainties in the system dynamics or models. Three kinds of typical approaches (e.g., closed-loop learning control, feedback control, and robust control) have been proved to be effective to solve these problems. This work presents a self-contained survey on the closed-loop and robust control of quantum systems, as well as a brief introduction to a selection of basic theories and methods in this research area, to provide interested readers with a general idea for further studies. In the area of closed-loop learning control of quantum systems, we survey and introduce such learning control methods as gradient-based methods, genetic algorithms (GA), and reinforcement learning (RL) methods from a unified point of view of exploring the quantum control landscapes. For the feedback control approach, the paper surveys three control strategies including Lyapunov control, measurement-based control, and coherent-feedback control. Then such topics in the field of quantum robust control as *H* ^*∞*^ control, sliding mode control, quantum risk-sensitive control, and quantum ensemble control are reviewed. The paper concludes with a perspective of future research directions that are likely to attract more attention.

## 1. Introduction

 Quantum mechanical systems model the dynamical evolution characterizing physical phenomena at atomic and molecular scales. Recent progress in theories and experiments has shown that such technologies as quantum information [1] and quantum control [2–4] have many advantages over their traditional counterparts. However, practical applications of quantum information technology are still confronted with some important technical difficulties such as the control of quantum systems in the presence of uncertainties or without explicit modeling information. Developing effective control theory and methods has been recognized as a solution to such difficulties. Some tools from classical control theory have been used to analyze and solve quantum control problems, among which we recall the most widely developed ones as follows.

*Closed-Loop Learning Control. *Closed-loop learning control has achieved great successes in controlling the laboratory quantum phenomena [5, 6], where the optimal open-loop control strategy is hard to design directly due to the incomplete knowledge of the system models or unexpected uncertainties.

*Feedback Control.* When a control is added to a quantum system, adjusting the control parameters according to instantaneous state of the system can make quantum control more pertinent and more effective, which will improve the control result with robustness and reliability. Feedback is such an effective control strategy in classical control theory as well as in quantum control theory, especially in the system with unpredictable disturbances in the evolution. During the past decades, different types of feedback control design methods have been studied for various applications [7–15].

*Robust Control.* In realistic environment, the quantum system is unavoidable to be subject to disturbances, uncertainties, and incomplete knowledge. These factors can all be viewed as uncertainties in the control field, in the Hamiltonian system, in the field-coupling coefficient (e.g., the dipole moment), and so forth and might affect the control results [16]. In order to achieve robustness in control method and to develop new insights into complicated quantum plants (such as quantum networks), it is desirable to apply classical robust control theory into quantum domain. Various kinds of robust control approaches [17–23] have been proposed, especially in the communities of control science.

All of these three kinds of approaches aim at optimizing the control performances for quantum systems that have no perfect models or are subject to uncertainties. But they are different from each other regarding specific motivations, methods, and applications. A brief comparison between these three kinds of approaches is shown in Table 1, and, in the following sections, we give a self-contained survey on these promising research areas and provide interested readers with a general idea for further studies.

The remaining of the paper is organized as follows. In Section 2, the closed-loop learning control problems of quantum systems are defined with the concept of quantum control landscape, and three kinds of closed-loop learning control methods (e.g., gradient-based, GA, and RL methods) are reviewed.  Section 3 introduced several feedback control approaches including Lyapunov control, measurement-based feedback control, and coherent-feedback control. Then such quantum robust control approaches as *H* ^*∞*^ control, sliding mode control, quantum risk-sensitive control, and quantum ensemble control are surveyed in Section 4. Conclusions and the authors' perspective on the future challenges in the associated fields are summarized in Section 5.

## 2. Closed-Loop Learning Control

 Learning control is an effective control method that can learn from experience and optimize the system performance by searching for the best control strategy in an iterative way. When applied to the control of quantum systems, as presented in [5], the closed-loop learning control procedure generally involves three elements: (i) a trial laser control input design, (ii) the laboratory generation of the control that is applied to the sample and subsequently observed for its impact, and (iii) a learning algorithm that considers the prior experiments and suggests the form of the next control input. It is clear that, for each trial of control, it is an open-loop control, while the control performance will be sent back to the learning algorithm to direct the optimization for the optimal control strategy.

The control objective is usually formulated as an optimal control problem by converting the problem into a problem of optimizing a functional of such control parameters as the quantum states, control inputs, control time, and so on. In order to systematically study the relationship between the time-dependent controls and the associated values of the objective functional, a notion of *quantum control landscape* [24, 25] is defined and related theories are also well developed. In this section, we will survey the area of closed-loop learning control from the point of view of quantum control landscape and introduce several practical and promising learning methods to explore the quantum control landscape, which includes the gradient-based methods, stochastic searching methods (e.g., genetic algorithm), and reinforcement learning methods.

### 2.1. Quantum Control Landscape: A Unified View for Closed-Loop Learning Control

**Figure 1:**
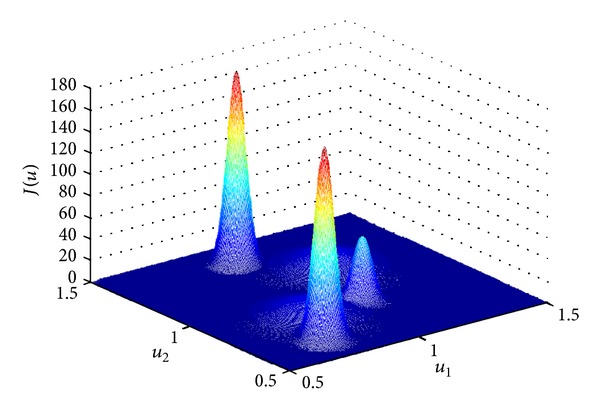
An example of quantum control landscape.

 In recent years, quantum control landscapes [25] have attracted more and more attention in the research field of quantum control. A control landscape is defined as the map between the time-dependent control Hamiltonian and associated values of the control performance functional. For example, as shown in Figure 1, the performance function *J*(*u*) is defined as the functional of the control strategy *u* = *u*_*i*_, *i* = 1,2,…, *M*, where *M* is a positive integer that indicates the number of the control variables (*M* = 2 for the case shown in Figure 1).

Quantum control aims to manipulate the dynamics of system evolution on the atomic and molecular scales, and most quantum control problems can be formulated as the maximization of an objective performance function. From a unified point of view, the closed-loop learning control is the approach of exploring a quantum control landscape to find the optimal control strategy where the objective function reaches its maximum or minimum. For the past decades, various algorithms have been proposed to explore the control landscapes for both theoretical studies and applications [24–29]. Most traditional learning methods can also be adopted to analyze or explore different kinds of control landscapes. In the next subsection, we survey these existing successful methods and classify them into three categories, that is, gradient-based methods, stochastic searching methods, and reinforcement learning methods.

### 2.2. Typical Learning Control Methods to Explore Quantum Control Landscape

#### 2.2.1. Gradient-Based Methods

 Gradient-based methods are one of the most important kinds of learning and optimization control methods for quantum systems [30, 31]. A well-developed gradient-based method called D-MORPH search algorithm is introduced in [32].

For most gradient-base methods, for example, we can introduce a time-like variable *s* to characterize different control strategies *u* ^(*s*)^(*t*). A gradient flow in the control space can be defined as

(1) du(s)(t)ds=−∇J(u(s)(t))=−(∂J∂u1(t),∂J∂u2(t),…,∂J∂uM(t)),

where ∇*J*(*u* ^(*s*)^(*t*)) is the gradient of *J* with respect to the control strategy *u* ^(*s*)^(*t*).

Choosing an arbitrary control strategy *u* ^0^(*t*), *t* ∈ [0, *T*], we can find the optimal one using gradient flow by solving the following initial value problem:

(2) du(s)ds=−∇J(u(s)(t)),u(0)(t)=u0(t).

According to (2), generally, we can approach the optimal control strategy by a forward Euler method over the *s*-domain; that is,

(3) $$\begin{array}{c} u\left(s+\Delta s,t\right)=u\left(s,t\right)-\Delta s\nabla J\left(u^{\left(s\right)}\left(t\right)\right). \end{array}$$

It is clear that, for a quantum control problem, the gradient-based methods are most likely effective provided that (i) we can get the gradient easily and (ii) there are no traps on the control landscape (otherwise, the learning process may get into the traps and cannot reach the maxima). Fortunately, as argued in [5, 33], it is surprising that, under certain conditions, most of the control landscapes are trap free, and it is easy to find the optimal solution for controlling most of the quantum phenomena. But for more complex tasks, we cannot guarantee the previous conditions or the gradient is hard to acquire, and hence other global searching methods for the closed-loop learning control are necessary.

#### 2.2.2. Stochastic Searching Methods

 Most of the stochastic searching methods are global searching methods and can step over traps of local maxima. One of the most widely used methods is genetic algorithm (GA), which has also achieved great success in the closed-loop learning control of laboratory quantum systems.

In the early 1990s, Judson and Rabitz [34] use a GA learning procedure to direct the production of pulses with a laboratory measurement device. Thereafter, GA methods have been widely applied to various quantum control problems. For example, in [35], an evolutionary algorithm is applied to femtosecond pulse shaping in optimal control experiments. Tsubouchi and Momose [36] use the GA algorithm to optimize the pulse shape for rovibrational wave-packet manipulation. Atabek et al. [37] use evolutionary algorithms for the optimal laser control of molecular orientation. The control and optimization prospects in the frequency domain are also studied theoretically using GA and shaping Fourier-limited pulses [38].

For more details about stochastic learning control methods like GA, for the laboratory closed-loop learning control, please refer to [6], where these methods are discussed within the concept of experimental adaptive feedback control (AFC).

#### 2.2.3. Reinforcement Learning Methods

 Reinforcement learning (RL) [39] is an active area of machine learning and has been extensively applied to traditional control problems ranging from operations research to robotics [40, 41]. Compared with other learning methods, RL is a learning technique based on trial and error and is a more general learning approach that can learn from experience and show great adaptability through an iterative way. That is to say, RL involves approximating solutions to stochastic optimal control problems under the condition of incomplete knowledge of the system, where the feedback for the closed loop is an imprecise value called reward or penalty. So RL methods are also suitable for control design of quantum systems [18] where it is difficult to get a good model or the searching problems are too complex to solve with computational efficiency.

On the other hand, the quantum characteristics also have inspired new algorithms for traditional RL methods. Dong et al. [42] proposed a new learning paradigm called quantum reinforcement learning (QRL) which fuses quantum computation with RL. In their study, the states {*s*_*i*_} or actions {*a*_*i*_} in traditional RL are denoted as the corresponding orthogonal quantum states and are called the eigen states {|*s*_*i*_〉} or eigen actions {|*a*_*i*_〉} in QRL. Here the action *a*_*i*_ represents the possible operation (or control) that can accomplish the state transition between two states. The state *s*_*i*_ can represent |*ϕ*_*l*_*i*__〉 and the action *a*_*i*_ represents the control function that can drive |*ϕ*_*l*_*i*__〉 to |*ϕ*_*l*_*i*+1__〉. This kind of representation with a parallel updating mechanism can speed up the learning process and improve the learning performance as well. Compared with the traditional RL, the QRL algorithm learns faster, its convergence range is much larger, and the learning rate is much easier to tune. QRL has been successfully applied for incoherent control of quantum systems [18]. Other quantum-inspired RL methods have also been studied for traditional control problems [43, 44].

Due to the strong learning and adaptive abilities, RL-based methods are promising for the exploration of quantum control landscapes and can be used for those quantum control problems where gradient-based or GA methods cannot work well. Thus the gradient-based methods (local search methods), GA methods (stochastic global search methods), and RL methods (global search methods but sometimes use the direction of gradient-like rewards) constitute three typical and different searching methods to explore the quantum control landscapes.

## 3. Feedback Control of Quantum Systems

 Quantum feedback control includes two central steps, that is, getting the information from the system and adding the control to the quantum system. The information of the quantum system can be obtained by two ways, precisely calculating the evolution of the system or fetching it from the quantum systems by some methods such as measurement. The former method is limited since a quantum system may have many unexpected affections during its evolution, while for the latter method, the measurement on a quantum system will unavoidably influence the states of the measured quantum system, making the situation more complex when applying feedback to quantum systems.

For a system with predictable state in the system, one can easily design the control field according to the instantaneous state of the system, and quantum Lyapunov control theory is such a kind of quantum control methods which obtain the message by exact simulation of the system. Actually, quantum Lyapunov control theory is only a feedback design of open-loop control theory, based on the artificial simulation of the system; thus it cannot be used in the case of unknown initial states or in the presence of unpredictable disturbance to the system.

There are two strategies for feedback control of quantum systems, measurement-based feedback, and coherent-feedback quantum control. The former strategy is to measure some quantum observable or signals of the system and to use and process the measurement results in a controller to produce a classical control signal that drives a suitable actuator, such as a laser beam or a potential well, which exerts direct influence on the quantum system to be controlled. The latter strategy is to use another quantum system without measurement, a full quantum controller, and connect it with the quantum system to be controlled in a feedback loop.

In the next three subsections, we will give a detailed survey of the above mentioned Lyapunov, measurement-based, and coherent quantum feedback control theories.

### 3.1. Feedback-Designed Open-Loop Quantum Control: Lyapunov Control

Quantum Lyapunov control uses feedback design to construct control fields but applies the fields into quantum systems in an open-loop way. It has been proposed as a good candidate for quantum state transfer [45, 46] and provides us with a simple way to design control fields without measurement and feedback ([47–57]).

Lyapunov function of quantum states is the central concept in quantum Lyapunov control theory; a function *V* is specified to design time-varying control fields. The system with state *ρ* converges to the target state given by *V* which monotonically decreases (or increases) to its minimum (or maximum), which is an observation *P* of the dynamics that are closely related to some property of target states,

(4) $$\begin{array}{c} V\left(\rho \right)=\mathrm{Tr}\left(P\rho \right), \end{array}$$

and *V* just stands for the distance between the present state and the target state. Then let the derivation of the Lyapunov function $\dot{V}<0$, which leads to the evolution of the system to the target state.

Assume that a closed quantum system with the free Hamiltonian *H*_0_ and the time-dependent control Hamiltonian *H*_*c*_(*t*) can be described by the following Liouville equation

(5) $$\begin{array}{c} \frac{d\rho \left(t\right)}{dt}=-i\left[H_{0}+H_{c}\left(t\right),\rho \left(t\right)\right]. \end{array}$$

Then the time derivative of the Lyapunov function can be calculated to design the control fields. By requiring

(6) $$\begin{array}{c} \dot{V}=\mathrm{Tr}\left(-iP\left[H_{0}+H_{c}\left(t\right),\rho \left(t\right)\right]\right)<0, \end{array}$$

one can work out the requirement of the parameter of the control Hamiltonian *H*_*c*_(*t*). Since the previous requirement may not completely determine the parameter, one can also find some further constraints to improve the control efficiency [57]. In essence, Lyapunov control uses the information of the system by simulation of the system, and it is a kind of feedback design control strategies.

This theory can be easily extended to the open quantum systems, such as the systems determined by

(7) $$\begin{array}{c} \frac{d\rho \left(t\right)}{dt}=-i\left[H_{0}+H_{c}\left(t\right),\rho \left(t\right)\right]+ℒ\left(\rho \left(t\right)\right), \end{array}$$

to study the control in open quantum systems.

In recent years, quantum Lyapunov control has been used to transfer quantum states [45, 46], to drive the states of open quantum system into the decoherence free subspaces [53], and to control the states in the decoherence free subspaces [54]. Also, Lyapunov control can be used to control the entanglement of the quantum systems [48, 52].

### 3.2. Measurement-Based Feedback Control

Measurement-based feedback (MFC) uses the measurement results to produce a classical control signal to drive a suitable actuator which exerts direct influence on the quantum system to be controlled [4]. During the MFC process, one can perform measurement on the system to get some information of the system state and then design the control law based on the estimation of the state. The system to be controlled is a quantum system, while the controller may be quantum, classical, or a quantum-classical hybrid. Different from the classical feedback control process, which can obtain the information of the system easily without disturbing it, the collapse of quantum state under the measurement process makes the problem of quantum systems rather complex.

*Markovian Quantum Feedback.* The general theory of quantum-limited feedback for continuously monitored systems was presented by Wiseman and Milburn, based on quantum measurement theory and on Hamiltonian system bath interactions [11, 58]. They considered the instantaneous feedback of some measured photocurrent (homodyne detection) onto the dynamics of a quantum system, and the master equation for the resulting evolution was Markovian; that is, the measurement record is immediately fed back into the system to alter the system dynamics and may then be forgotten, while any time delay is ignored and a memoryless controller is assumed. Hence, the equation describing the resulting evolution is a Markovian master equation. This kind of feedback has already been used to reduce laser noise below the shot-noise level [59] and also has been used in many aspects of physical problems, such as the control of quantum qubits [60] and quantum entanglement [61–64]. In case the feedback delay cannot be ignored, the feedback Hamiltonian must include a delay parameter. Time delay effect of the measurement was investigated in [65].

*Bayesian Feedback Method.* Doherty and Jacobs presented a formulation of feedback in quantum systems in which the best estimates of the dynamical variables are obtained continuously from the measurement record and fed back to control the system [7, 12]. They considered some arbitrary functional of the entire history of the measurement results that can be used to alter the system evolution. The resulting equation dynamics of the system are then non-Markovian. However, the dynamics of the system and controller remain Markovian, and this is completely analogous to the situation in classical control theory. In Bayesian quantum feedback, the control process is also divided into two steps involving state estimation and feedback control. Due to the fact that, in linear systems, the estimation process may be modeled by its classical analogue, Kalman filtration and classical linear quadratic Gaussian (LQG) control theory may be applied to quantum feedback by estimation. For Bayesian quantum feedback, it uses a more general form of control Hamiltonian with more information from the measurement.

It has been compared in [14] the Bayesian and Markovian feedback quantum controls, where it was proved that Bayesian feedback is never inferior, and since it uses more information, it is usually superior to Markovian feedback. However, it would be far more difficult to implement than Markovian feedback and it loses its superiority when obvious simplifying approximations are made. Thus, it is not clear which form of feedback would be better in the face of inevitable experimental imperfections. Bayesian feedback has also been used in many aspects of systems, such as the preparation of quantum states [66], and quantum error correction [67, 68].

*Weak Measurement and Nondemolition Measurement.* Making as little influence as possible during the measurement process is important to minimize disturbance to the system to be controlled. Weak measurement makes it possible to modify the evolution continuously via Hamiltonian feedback, where the Hamiltonian feedback applied to the system depends on the measurement record [69], and it can also be modeled by a stochastic master equation by introducing an ancilla system weakly coupled to the system of interest. Weak measurements and local feedback can be used to control the generation of entanglement between two qubits [70]. Motivated by the proposal of Brańczyk et al. [71], experimentally exploring the use of weak measurement [72] in feedback control on a photonic polarization qubit is given in [73], as well as in the control of nonlinear quantum systems [74]. Quantum nondemolition measurement preserves the integrity of the system and the value of the measured observable, which is best thought of as the ideal quantum projective measurement. Nevertheless, nondemolition does not mean that the state of the system haas no wave collapse, and it is extremely difficult to carry out experimentally [75].

Although measurement-based feedback control is effective in many quantum control systems, its drawbacks are also evident. Firstly, measuring a quantum system almost inevitably disturbs it. Even a nondemolition measurement that leaves the system in the state in which it was measured still typically alters the states of the system prior to the measurement [76, 77]. After fluorescence determines whether the ion is in its ground state or excited state, the initial quantum coherence between those states is irrevocably lost. Secondly, the information from the measurement is stochastic because a result of the measurement of the system jumps to one state or another probabilistically. Although the ability to apply coherent operations conditioned on the results of measurements allows the controller to compensate for the probabilistic nature of their results, the introduction of stochastic effects significantly complicates the control process. Furthermore, the measurement-based feedback is limited by its information processing speed that has to be kept up with the evolution of the system dynamics, and it cannot be used in most solid state systems whose time scales range from picoseconds to nanoseconds.

### 3.3. Coherent-Feedback Control

Coherent-feedback quantum control uses another quantum system as a full quantum controller and connects it with the quantum system to be controlled in a feedback loop; that is, the feedback controller itself is a quantum system, and the control operations consist of unitary transformations. This is greatly different from Markovian and Bayesian quantum feedback controls where the feedback information from measurement results is classical information and the feedback controller is a classical controller.

Since this control uses full quantum information of the system, it can perform a number of tasks that controllers using a classical information feedback loop cannot [15]. Compared with the measurement-based feedback control, coherent-feedback control does not involve measurement, avoiding the introduction of excess measurement noise, while the controller and the system plant can be both quantum systems and are coherently connected. By coherent-feedback control, one can use coherent feedback to guide a quantum system from an unknown initial state to a desired final state without destroying the initial state. In addition, a controller can use a quantum feedback loop to drive a quantum system to a target state that is entangled with another quantum system, while entanglement is a nonlocal quantum phenomenon that cannot be created by controllers using classical feedback loops.

The very successful noise-reducing controllers, the *H* ^*∞*^ and the linear quadratic Gaussian (LQG) controllers, have natural coherence control analogues [17, 78, 79]. By basic principles of linear quantum stochastic control theory, it has been presented that optimal and robust design of quantum coherent-feedback loops can be accomplished using sophisticated methods of system engineering [17], and an experimental implementation of coherent-feedback quantum control with optical resonators as the dynamical systems and laser beams as the coherent disturbance and feedback signals has been presented [80]. The experiments of coherent-feedback control in optical field squeezing are proposed in [81], and it was also applied to many other interesting problems, such as cooling quantum oscillator [82], spontaneous switching suppression [83], multipartite quantum entanglement generation [84], and producing optical quantum gates in a four-wave mixing process [85].

Traditional coherence feedback control was established for the Markovian environment. Recently, the non-Markovian coherence feedback control was presented [86]. However, in coherence feedback control, the controller itself will cause quantum decoherence to the controlled system even though it coherently entangles with the system [87]; thus, whether the coherent feedback is better than the open-loop control for quantum control systems needs to be investigated in depth [88].

## 4. Robust Control

 A general formalism of quantum robust optimal control problem was given in [16], which pointed out that to design a control field that achieves the best objective functional under possible worst uncertainties is in essence a minimax problem. Reference [16] also provided a method to calculate the worst possible disturbance to the control process and to design a corresponding robust optimal control field. Another noticeable early attempt to apply robust control theory in quantum field is [89], where the small gain theorem was extended to analyze the stability of quantum feedback networks. Later, different robust control tools were systematically introduced into the quantum domain, which formulated the early development of quantum robust control.

### 4.1. *H* ^*∞*^ Control of Quantum Systems

 For several typical classes of noncommutative linear stochastic systems with many interesting examples in quantum technology, *H* ^*∞*^ *control theory* was introduced to obtain robust controllers and developed for diverse situations and requirements.

Take the class of linear noncommutative stochastic systems in [17] for example, which encompasses some quantum and classical systems:

(8) dx(t)=Ax(t)dt+Bdω(t),  x(0)=x0,dy(t)=Cx(t)dt+Ddω(t),

where *A*, *B*, *C*, and *D* are, respectively, real **R** ^*n*×*n*^, **R** ^*n*×*n*_*ω*_^, **R** ^*n*_*y*_×*n*^, and **R** ^*n*_*y*_×*n*_*ω*_^ matrices with *n*, *n*_*ω*_, and*n*_*y*_ all positive integers and *x*(*t*) = [*x*_1_(*t*),…, *x*_*n*_(*t*)]^*T*^ is a vector of self-adjoint possibly noncommutative system variables, whose initial value *x*_0_ consists of operators satisfying the commutation relations

(9) $$\begin{array}{c} \left[x_{j}\left(0\right),x_{k}\left(0\right)\right]=2i\Theta_{jk},\;j,k=1,\ldots ,n, \end{array}$$

where [*A*, *B*] = *AB* − *BA* is the commutation operator, Θ_*jk*_ are components of the real antisymmetric matrix Θ, and *i* is the imaginary unit. *x*_0_ is also assumed to be Gaussian with density operator *ρ*. The vector quantity *ω* describes the input signals and is assumed to have the decomposition

(10) $$\begin{array}{c} d\omega \left(t\right)=\beta_{\omega }\left(t\right)dt+d\tilde{\omega }\left(t\right), \end{array}$$

where *β*_*ω*_(*t*) is a self-adjoint, adapted process (see [90, 91]). The noise part of *ω*(*t*) is $\tilde{\omega }(t)$, a vector of self-adjoint quantum noises with Ito table

(11) $$\begin{array}{c} d\tilde{\omega }\left(t\right)d{\tilde{\omega }}^{T}\left(t\right)=F_{\tilde{\omega }}dt, \end{array}$$

where $F_{\tilde{\omega }}$ is a nonnegative Hermitian matrix (see [91, 92]). For more detailed description and assumptions and the physical realisability of this class of systems, one can refer to [17].

The *H* ^*∞*^ controller synthesis problem for the class of systems described by (8)–(11) was first formulated and solved in [17]. Furthermore, this quantum *H* ^*∞*^ control problem was extended to a time-varying version, and the corresponding solution was obtained by a dynamic game approach in [93]. For the same plant, the finite horizon dynamic game theory approach was applied in [94], and the solving process was proved equivalent to solving a corresponding deterministic continuous-time problem with imperfect state information. The finite horizon *H* ^*∞*^ control problem in [94] was then extended to the case of delayed measurements in [95].

To simplify the deduction process and obtain more profound results, a more special class of linear quantum systems was considered in [79], which proposed a robust controller designing method probably more easy to implement experimentally.

### 4.2. Sliding Mode Control of Quantum Systems

*Sliding mode control (SMC)* approach is a useful robust control tool in classical control theory and industrial applications, especially for nonlinear systems. Since many quantum systems evolve with nonlinear equations, SMC is therefore supposed to be capable of controlling some quantum phenomena [96, 97].

Reference [98] applied the SMC control method into quantum systems. Similar to the classical theory, quantum sliding mode is a system state where the system has some desirable features, such as robustness to a class of uncertainties, and features brought by eigenstates, features brought by invariant state subspaces. Once the sliding mode is selected, one needs to design control laws that can drive the system onto its sliding mode and keep the system on it, which were designed in detail by combining unitary control and periodic projective measurements in [98].

In [99], a sliding mode design method for two-level quantum systems with bounded uncertainties was proposed. The uncertainties were assumed to take the form of perturbations in the Hamiltonian, and the controller design method used the Lyapunov methodology and periodic projective measurements. These results were extended in [100], where the effect of uncertainties in driving the system state back to the sliding mode domain from outside was considered, and the measurement periods were modified when considering uncertainties described as perturbations in the free Hamiltonian. In [101], a sampled-data design approach for decoherence control of a single qubit with operator errors was proposed using a sliding mode domain concept as the required control performance.

Though sliding mode control approach was introduced into quantum systems, the appropriate combination of the essential characters of these two focuses is still worth digging. Furthermore, one may consider extending sliding mode control to open quantum systems and applying other branches of classical nonlinear control theories into the quantum domain.

### 4.3. Quantum Risk-Sensitive Control

 As a modification of the common integral form of criterion, or the so-called a risk-neutral criterion, a *risk-sensitive criterion* takes the form of an exponential function, which results in the close connections between robust control and *risk-sensitive control* [102–104]. For example, risk-sensitive control is anticipated to be useful in designing robust controllers [105]. Reference [106] formulated a risk-sensitive optimal control problem for quantum systems, obtained a solution using dynamic programming, and briefly discussed the robustness properties of the risk-sensitive controllers. Reference [107] considered a risk-sensitive optimal control problem for continuously monitored open quantum systems within the framework of quantum Langevin equations and solved the problem with quantum stochastic calculus and dynamic programming. Reference [105] collected related research and systematically illustrated a quantum risk-sensitive control problem and the corresponding dynamic-programming solution. At the end of [105], the author proposed several developing directions for quantum risk-sensitive control, which include theoretical development, practical applications in quantum field, and the exploration of robustness properties.

*Filtering* aims to extract information from noisy signals and is inherently connected with robust control, which therefore forms *robust estimation*. Guaranteed-cost filtering and risk-sensitive filtering are two branches of robust estimation, which are quite promising to be extended into quantum theory. Reference [108] obtained a quantum version of the guaranteed-cost filter and showed its unique robustness character compared with optimal Kalman filter and risk-sensitive observer. Reference [23] studied a quantum risk-sensitive estimation problem and analyzed robustness properties of the filter under a discrete approximation model of the aimed quantum system. More systematic work within the associated topics remains to be done.

### 4.4. Quantum Ensemble Control

*Ensemble control* means controlling a continuum of dynamical systems with different values of parameters characterizing the system dynamics by using the same control signal. Ensemble control derives from the manipulation of an ensemble of nuclear spins in nuclear magnetic resonance (NMR) spectroscopy and imaging (MRI), where one often needs to develop external excitations that can simultaneously steer the ensemble of systems with variations in their internal parameters from a fiducial state to a target state [109]. Here we view the difference in parameters as system uncertainties. Hence, ensemble control forms a new systematic branch of robust control.

A fundamental question in quantum ensemble control is controllability, which determines whether the control function that transfers the system from initial states to desired target states can exist. Reference [110] introduced the notion of *simultaneous controllability*; that is, all individuals in the system are simultaneously controllable, and generalized controllability criteria for decomposable systems. References [111, 112] formally proposed the definition of *ensemble controllability* for quantum systems described by Bloch equations depending continuously on a finite number of scalar parameters and with a finite number of control inputs and analyzed ensemble controllability and optimal control of linear time-invariant systems. Ensemble controllability concerns finding open-loop controls to compensate for the dispersion in element parameters. Reference [113] cast the design of control pulses as an optimal ensemble control problem and introduced a multidimensional pseudospectrum-based solution, whose convergence was shown in [114]. Reference [115] studied the controllability of an ensemble of general finite-dimensional time-varying linear systems and gave necessary and sufficient conditions, which is in connection with singular values of the operator characterizing the system dynamics. Reference [116] introduced a universal numerical method based on the singular value decomposition to approximate optimal ensemble control problems.

Furthermore, since ensemble is originally a notion in quantum statistics, one may anticipate introducing tools and methods in quantum statistical mechanics into quantum ensemble control to give new in sights and approaches.

## 5. Conclusions and Discussions

 Manipulating system dynamics at the quantum scale is full of challenges for both theoretical and laboratory researchers. Closed-loop and robust control approaches are of most importance to deal with uncertainties and incomplete knowledge about the system dynamics or unexpected disturbances. To conclude this paper, we briefly discuss some open problems and promising research directions as follows.

Learning skills is very important for the control design of quantum systems where no good solutions can be easily obtained from a specific model. Although the closed-loop learning control approach for controlling quantum phenomena has been well developed since the early 1990s, more effective learning theories and algorithms need to be further explored. The experts from different fields such as quantum physics, chemical physics, control theory, computer science, and artificial intelligence need to cooperate on this exciting research area.

Feedback control is one of the most important control strategies for traditional control problems. Almost all the practical industrial control systems use feedback controllers such as PID controllers. In the feedback control approach, the deviations between the measured variable and a set point are fed back to the controller to generate appropriate control actions. When we apply feedback control methods for the quantum control systems, two problems are unavoidable, that is, the problems of measurement and time scale. Quantum state measurement is difficult and much more complex than its counterpart of traditional control systems. Time scale is another nontrivial issue for the control of quantum systems since the feedback signals are always lagging. The feedback control design needs to incorporate the time delay of the feedback signal and satisfy the time scale of the controlled quantum systems.

For practical applications, robustness is an important aspect for the design of controllers, especially for quantum systems that are subject to various kinds of uncertainties and are more fragile. The existing results mainly focus on certain kinds of quantum systems with specific models, and experiments temporarily fall behind the development of theory. In the future, more general and systematic approaches of robust control need to be developed for more general kinds of uncertainties which exist in practical applications.

## Figures and Tables

**Table 1:** Closed-loop and robust control approaches for quantum systems.

	Motivations	Typical methods	Applications
Closed-loop learning control	Direct the control results and procedures in an iteratively learning way	(1) Gradient-based methods (2) Stochastic searching (GA) (3) RL methods	Controlling laboratory quantum phenomena with incomplete knowledge or unexpected uncertainties, for example, optimal laser control design.
Feedback control	Adjust control parameters according to instantaneous feedback information	(1) Lyapunov control (2) Measurement-based control (3) Coherent-feedback control	Quantum state transition control, entanglement control, design of quantum gates, and so forth.
Robust control	Design control to achieve the best objective functional under the possible worst uncertainties	(1) *H* ^*∞*^ control (2) Sliding mode control (3) Risk-sensitive control (4) Quantum ensemble control	Control design for quantum systems that are fragile and are subject to various kinds of uncertainties.

## References

[1] Nielsen MA, Chuang IL (2000). *Quantum Computation and Quantum Information*.

[2] Altafini C, Ticozzi F (2012). Modeling and control of quantum systems: an introduction. *IEEE Transactions on Automatic Control*.

[3] Dong D, Petersen IR (2010). Quantum control theory and applications: a survey. *IET Control Theory and Applications*.

[4] Wiseman HM, Milburn GJ (2010). *Quantum Measurement and Control*.

[5] Rabitz H, De Vivie-Riedle R, Motzkus M, Kompa K (2000). Whither the future of controlling quantum phenomena?. *Science*.

[6] Brif C, Chakrabarti R, Rabitz H (2010). Control of quantum phenomena: past, present and future. *New Journal of Physics*.

[7] Doherty AC, Habib S, Jacobs K, Mabuchi H, Tan SM (2000). Quantum feedback control and classical control theory. *Physical Review A*.

[8] van Handel R, Stockton JK, Mabuchi H (2005). Feedback control of quantum state reduction. *IEEE Transactions on Automatic Control*.

[9] Mirrahimi M, Van Handel R (2007). Stabilizing feedback controls for quantum systems. *SIAM Journal on Control and Optimization*.

[10] Yanagisawa M, Kimura H (2003). Transfer function approach to quantum control-part I: dynamics of quantum feedback systems. *IEEE Transactions on Automatic Control*.

[11] Wiseman HM, Milburn GJ (1993). Quantum theory of optical feedback via homodyne detection. *Physical Review Letters*.

[12] Doherty AC, Jacobs K (1999). Feedback control of quantum systems using continuous state estimation. *Physical Review A*.

[13] Zhang J, Wu R-B, Li C-W, Tarn T-J (2010). Protecting coherence and entanglement by quantum feedback controls. *IEEE Transactions on Automatic Control*.

[14] Wiseman HM, Mancini S, Wang J (2002). Bayesian feedback versus Markovian feedback in a two-level atom. *Physical Review A*.

[15] Lloyd S (2000). Coherent quantum feedback. *Physical Review A*.

[16] Zhang H, Rabitz H (1994). Robust optimal control of quantum molecular systems in the presence of disturbances and uncertainties. *Physical Review A*.

[17] James MR, Nurdin HI, Petersen IR (2008). H∞ control of linear quantum stochastic systems. *IEEE Transactions on Automatic Control*.

[18] Dong DY, Chen C, Tarn T-J, Pechen A, Rabitz H (2008). Incoherent control of quantum systems with wavefunction-controllable subspaces via quantum reinforcement learning. *IEEE Transactions on Systems, Man, and Cybernetics B*.

[19] Dong D, Lam J, Tarn TJ (2009). Rapid incoherent control of quantum systems based on continuous measurements and reference model. *IET Control Theory and Applications*.

[20] Altafini C (2007). Feedback stabilization of isospectral control systems on complex flag manifolds: application to quantum ensembles. *IEEE Transactions on Automatic Control*.

[21] Pravia MA, Boulant N, Emerson J (2003). Robust control of quantum information. *Journal of Chemical Physics*.

[22] Chen C, Dong D, Lam J, Chu J, Tarn TJ (2012). Control design of uncertain quantum systems with fuzzy estimators. *IEEE Transactions on Fuzzy Systems*.

[23] Yamamoto N, Bouten L (2009). Quantum risk-sensitive estimation and robustness. *IEEE Transactions on Automatic Control*.

[24] Rabitz HA, Hsieh MM, Rosenthal CM (2004). Quantum optimally controlled transition landscapes. *Science*.

[25] Chakrabarti R, Rabitz H (2007). Quantum control landscapes. *International Reviews in Physical Chemistry*.

[26] Rabitz H, Hsieh M, Rosenthal C (2005). Landscape for optimal control of quantum-mechanical unitary transformations. *Physical Review A*.

[27] Rothman A, Ho T-S, Rabitz H (2006). Exploring the level sets of quantum control landscapes. *Physical Review A*.

[28] Shen Z, Hsieh M, Rabitz H (2006). Quantum optimal control: hessian analysis of the control landscape. *Journal of Chemical Physics*.

[29] Wu R, Rabitz H, Hsieh M (2008). Characterization of the critical submanifolds in quantum ensemble control landscapes. *Journal of Physics A*.

[30] Roslund J, Rabitz H (2009). Gradient algorithm applied to laboratory quantum control. *Physical Review A*.

[31] Strasfeld DB, Shim S-H, Zanni MT (2007). Controlling vibrational excitation with shaped Mid-IR pulses. *Physical Review Letters*.

[32] Rothman A, Ho T-S, Rabitz H (2005). Observable-preserving control of quantum dynamics over a family of related systems. *Physical Review A*.

[33] Pechen AN, Tannor DJ (2011). Are there traps in quantum control landscapes?. *Physical Review Letters*.

[34] Judson RS, Rabitz H (1992). Teaching lasers to control molecules. *Physical Review Letters*.

[35] Zeidler D, Frey S, Kompa K-L, Motzkus M (2001). Evolutionary algorithms and their application to optimal control studies. *Physical Review A*.

[36] Tsubouchi M, Momose T (2008). Rovibrational wave-packet manipulation using shaped midinfrared femtosecond pulses toward quantum computation: optimization of pulse shape by a genetic algorithm. *Physical Review A*.

[37] Atabek O, Dion CM, Ben Haj Yedder A (2003). Evolutionary algorithms for the optimal laser control of molecular orientation. *Journal of Physics B*.

[38] Gollub C, De Vivie-Riedle R (2008). Theoretical optimization and prediction in the experimental search space for vibrational quantum processes. *Physical Review A*.

[39] Chen C, Dong D, Li H-X, Tarn T-J (2011). Hybrid MDP based integrated hierarchical Q-learning. *Science China Information Sciences*.

[40] Chen C, Li H-X, Dong D (2008). Hybrid control for robot navigation—a hierarchical Q-learning algorithm. *IEEE Robotics and Automation Magazine*.

[41] Chen C, Dong D (2010). Grey system based reactive navigation of mobile robots using reinforcement learning. *International Journal of Innovative Computing, Information and Control*.

[42] Dong D, Chen C, Li H, Tarn T-J (2008). Quantum reinforcement learning. *IEEE Transactions on Systems, Man, and Cybernetics B*.

[43] Chen CL, Dong DY, Chen ZH (2006). Quantum computation for action selection using reinforcement learning. *International Journal of Quantum Information*.

[44] Dong D, Chen C, Chu J, Tarn T-J (2012). Robust quantum-inspired reinforcement learning for robot navigation. *IEEE/ASME Transactions on Mechatronics*.

[45] Grivopoulos S, Bamieh B Lyapunov-based control of quantum systems.

[46] Vettori P On the convergence of a feedback control strategy for multilevel quantum systems.

[47] Mirrahimi M, Rouchon P, Turinici G (2005). Lyapunov control of bilinear Schrödinger equations. *Automatica*.

[48] Wang X, Schirmer SG (2010). Analysis of Lyapunov method for control of quantum states. *IEEE Transactions on Automatic Control*.

[49] Kuang S, Cong S (2008). Lyapunov control methods of closed quantum systems. *Automatica*.

[50] Coron J-M, Grigoriu A, Lefter C, Turinici G (2009). Quantum control design by Lyapunov trajectory tracking for dipole and polarizability coupling. *New Journal of Physics*.

[51] Beauchard K, Coron JM, Mirrahimi M, Rouchon P (2007). Implicit Lyapunov control of finite dimensional Schrödinger equations. *Systems and Control Letters*.

[52] Wang X, Schirmer SG (2009). Entanglement generation between distant atoms by Lyapunov control. *Physical Review A*.

[53] Yi XX, Huang XL, Wu C, Oh CH (2009). Driving quantum systems into decoherence-free subspaces by Lyapunov control. *Physical Review A*.

[54] Wang W, Wang LC, Yi XX (2010). Lyapunov control on quantum open systems in decoherence-free subspaces. *Physical Review A*.

[55] Yi XX, Wu SL, Wu C, Feng XL, Oh CH (2011). Time-delay effects and simplified control fields in quantum Lyapunov control. *Journal of Physics B*.

[56] Yi XX, Cui B, Wu C, Oh CH (2011). Effects of uncertainties and errors on a Lyapunov control. *Journal of Physics B*.

[57] Hou SC, Khan MA, Yi XX, Daoyi Dong, Petersen RL (2012). Optimal Lyapunov-based quantum control for quantum systems. *Physical Review A*.

[58] Wiseman HM (1994). Quantum theory of continuous feedback. *Physical Review A*.

[59] Machida S, Yamamoto Y (1986). Observation of sub-poissonian photoelectron statistics in a negative feedback semiconductor laser. *Optics Communications*.

[60] Mabuchi H, Zoller P (1996). Inversion of quantum jumps in quantum optical systems under continuous observation. *Physical Review Letters*.

[61] Wang J, Wiseman HM, Milburn GJ (2005). Dynamical creation of entanglement by homodyne-mediated feedback. *Physical Review A*.

[62] Stevenson RN, Hope JJ, Carvalho ARR (2011). Engineering steady states using jump-based feedback for multipartite entanglement generation. *Physical Review A*.

[63] Carvalho ARR, Reid AJS, Hope JJ (2008). Controlling entanglement by direct quantum feedback. *Physical Review A*.

[64] Stevenson RN, Hope JJ, Carvalho ARR (2011). Engineering steady states using jump-based feedback for multipartite entanglement generation. *Physical Review A*.

[65] Ge SS, Vu TL, Lee TH (2012). Quantum measurement-based feedback control: a nonsmooth time delay control approach. *SIAM Journal on Control and Optimization*.

[66] Ruskov R, Korotkov AN (2002). Quantum feedback control of a solid-state qubit. *Physical Review B*.

[67] Ahn C, Doherty AC, Landahl AJ (2002). Continuous quantum error correction via quantum feedback control. *Physical Review A*.

[68] Sarovar M, Ahn C, Jacobs K, Milburn GJ (2004). Practical scheme for error control using feedback. *Physical Review A*.

[69] Combes J, Jacobs K (2006). Rapid state reduction of quantum systems using feedback control. *Physical Review Letters*.

[70] Hill C, Ralph J (2008). Weak measurement and control of entanglement generation. *Physical Review A*.

[71] Brańczyk AM, Mendona PEMF, Gilchrist A, Doherty AC, Bartlett SD (2007). Quantum control of a single qubit. *Physical Review A*.

[72] Aharonov Y, Vaidman L (1990). Properties of a quantum system during the time interval between two measurements. *Physical Review A*.

[73] Gillett GG, Dalton RB, Lanyon BP (2010). Experimental feedback control of quantum systems using weak measurements. *Physical Review Letters*.

[74] Zhang J, Liu Y-X, Wu R-B, Li C-W, Tarn T-J (2010). Transition from weak to strong measurements by nonlinear quantum feedback control. *Physical Review A*.

[75] Braginsky VB, Khalili FY (1996). Quantum nondemolition measurements: the route from toys to tools. *Reviews of Modern Physics*.

[76] Braginsky VB, Vorontsov YI, Thorne KS (1980). Quantum nondemolition measurements. *Science*.

[77] Caves CM, Thorne KS, Drever RWP, Sandberg VD, Zimmermann M (1980). On the measurement of a weak classical force coupled to a quantum-mechanical oscillator. I. Issues of principle. *Reviews of Modern Physics*.

[78] Maalouf AI, Petersen IR Coherent H∞ control for a class of linear complex quantum systems.

[79] Maalouf AI, Petersen IR (2011). Coherent *H*
^*∞*^ control for a class of annihilation operator linear quantum systems. *IEEE Transactions on Automatic Control*.

[80] Mabuchi H (2008). Coherent-feedback quantum control with a dynamic compensator. *Physical Review A*.

[81] Iida S, Yukawa M, Yonezawa H, Yamamoto N, Furusawa A (2012). Experimental demonstration of coherent feedback control on optical field squeezing. *IEEE Transactions on Automatic Control*.

[82] Hamerly R, Mabuchi H (2012). Advantages of coherent feedback for cooling quantum oscillators. *IEEE Transactions on Automatic Letters*.

[83] Mabuchi H (2011). Coherent-feedback control strategy to suppress spontaneous switching in ultralow power optical bistability. *Applied Physics Letters*.

[84] Yan Z, Jia X, Xie C, Peng K (2011). Coherent feedback control of multipartite quantum entanglement for optical fields. *Physical Review A*.

[85] Zhou Z, Liu C, Fang Y (2012). Optical logic gates using coherent feedback. *Applied Physics Letters*.

[86] Xue SB, Wu RB, Zhang WM, Zhang J, Li CW, Tarn TJ (2012). Decoherence suppression via non-Markovian coherent feedback
control. *Physical Review A*.

[87] Xue F, Yu SX, Sun CP (2006). Quantum control limited by quantum decoherence. *Physical Review A*.

[88] Qi B, Guo L (2010). Is measurement-based feedback still better for quantum control systems?. *Systems and Control Letters*.

[89] D'Helon C, James MR (2006). Stability, gain, and robustness in quantum feedback networks. *Physical Review A*.

[90] Bouten L, Van Handel R, James MR (2007). An introduction to quantum filtering. *SIAM Journal on Control and Optimization*.

[91] Parthasarathy KR (1992). *An Introduction To Quantum stochastic Calculus*.

[92] Belavkin VP (1992). Quantum continual measurements and a posteriori collapse on CCR. *Communications in Mathematical Physics*.

[93] Maalouf AI, Petersen IR (2012). Time-varying *H*
^*∞*^ control for a class of linear quantum systems: a dynamic game approach. *Automatica*.

[94] Maalouf AI, Petersen IR Finite horizon H∞ control for a class of linear quantum systems: a dynamic game approach.

[95] Maalouf AI, Petersen IR Finite horizon H∞ control for a class of linear quantum measurement delayed systems: A dynamic game approach.

[96] Dong D, Petersen IR Variable structure control of uncontrollable quantum systems.

[97] Mendes RV, Man'ko VI (2003). Quantum control and the Strocchi map. *Physical Review A*.

[98] Dong D, Petersen IR (2009). Sliding mode control of quantum systems. *New Journal of Physics*.

[99] Dong D, Petersen IR (2012). Sliding mode control of two-level quantum systems. *Automatica*.

[100] Dong D, Petersen IR (2012). Notes on sliding mode control of twolevel quantum systems. *Automatica*.

[101] Dong D, Petersen IR, Rabitz H (2013). Sampled-data design for robust control of a single qubit. *Transactions on Automatic Control*.

[102] Doyle JC, Glover K, Khargonekar PP, Francis BA (1989). State-space solutions to standard H2 and H∞ control problems. *IEEE Transactions on Automatic Control*.

[103] Glover K, Doyle JC (1988). State-space formulae for all stabilizing controllers that satisfy an H∞-norm bound and relations to relations to risk sensitivity. *Systems and Control Letters*.

[104] James MR, Baras JS, Elliott RJ (1994). Risk-sensitive control and dynamic games for partially observed discrete-time nonlinear systems. *IEEE Transactions on Automatic Control*.

[105] D'Helon C, Doherty AC, James MR, Wilson SD Quantum risk-sensitive control.

[106] James MR (2004). Risk-sensitive optimal control of quantum systems. *Physical Review A*.

[107] James MR A quantum Langevin formulation of risk-sensitive optimal control. *Jounal of Optics B*.

[108] Yamamoto N (2006). Robust observer for uncertain linear quantum systems. *Physical Review A*.

[109] Li J-S, Khaneja N (2009). Ensemble control of bloch equations. *IEEE Transactions on Automatic Control*.

[110] Schirmer SG, Pullen ICH, Solomon AI (2005). Controllability of multi-partite quantum systems and selective excitation of quantum dots. *Journal of Optics B*.

[111] Li J-S, Khaneja N Ensemble controllability of the bloch equations.

[112] Li J-S, Khaneja N Ensemble control of linear systems.

[113] Ruths J, Li J-S (2011). A multidimensional pseudospectral method for optimal control of quantum ensembles. *Journal of Chemical Physics*.

[114] Ruths J, Li JS (2012). Optimal control of inhomogeneous ensembles. *IEEE Transactions on Automatic Control*.

[115] Li J-S (2011). Ensemble control of finite-dimensional time-varying linear systems. *IEEE Transactions on Automatic Control*.

[116] Li JS, Zlotnik A Synthesis of optimal ensemble controls for linear systems using the singular value decomposition.

